# Language processing in Internet use disorder: Task-based fMRI study

**DOI:** 10.1371/journal.pone.0269979

**Published:** 2022-06-24

**Authors:** Gergely Darnai, Gábor Perlaki, Gergely Orsi, Ákos Arató, Anna Szente, Réka Horváth, Eszter Áfra, Szilvia Anett Nagy, Norbert Kovács, Tamás Dóczi, József Janszky

**Affiliations:** 1 Department of Behavioural Sciences, Medical School, University of Pécs, Pécs, Hungary; 2 Department of Neurology, Medical School, University of Pécs, Pécs, Hungary; 3 MTA-PTE Clinical Neuroscience MR Research Group, Pécs, Hungary; 4 Pécs Diagnostic Centre, Pécs, Hungary; 5 Department of Neurosurgery, Medical School, University of Pécs, Pécs, Hungary; 6 Neurobiology of Stress Research Group, Szentágothai Research Centre, University of Pécs, Pécs, Hungary; 7 Department of Laboratory Medicine, Medical School, University of Pécs, Pécs, Hungary; Southwest University, CHINA

## Abstract

Internet use disorder (IUD) is generally conceptualized as a fast-growing behavioral addiction. Several structural and functional brain alterations have been revealed in this condition, but previous behavioral studies indicated that language systems may also be impaired. We used a silent word generation task to induce brain activation in Broca’s area and other parts of the language system. Blood-oxygen-level-dependent activation analysis and psychophysiological interaction analysis were applied to assess functional brain changes. IUD was measured by the Problematic Internet Use Questionnaire and two additional questions concerning usage time and subjective rating of addiction. According to our key findings, areas strongly related to the default mode network were altered in IUD during the task. Moreover, Broca’s area showed altered functional connectivity with other language network and occipital areas in IUD. These findings may address the neural background of decreased verbal fluency performance previously reported in the literature, and we emphasize that beside the brain’s reward and inhibitory control systems, the language system is the next candidate to be involved in the pathogenesis of IUD.

## Introduction

Internet use disorder (IUD) is defined as an excessive and prolonged Internet usage pattern that causes behavioral and cognitive problems and is generally conceptualized as a behavioral addiction [1]. It shows core components of addictive behaviors, including progressive loss of control over Internet use, obsessive thoughts of the Internet, and neglect of everyday activities, social life, and work performance [2]. Although Internet overuse has appeared as a new mental health concern, a consensus regarding diagnostic criteria and measures is still highly needed [3]. Most authors claim that it is important to distinguish between IUD and specific forms of Internet addiction such as Internet gaming, online shopping, or social media addictions, e.g. Montag et al. found that only social network addiction showed constant correlation with generalized IUD and other specific forms must be distinguished during scientific investigations [4].

Functional brain alterations related to IUD have previously been revealed. Significant differences were found in brain regions involved in cognitive control [5] and reward processing [6] (for a comprehensive review, see [7]). These studies investigated both general and specific IUD forms (including online gaming; the most widely investigated form) and merely used task-related and resting-state functional magnetic resonance imaging (fMRI). Through these studies, the physiology and pathogenesis of IUD is now much more exposed, however, besides the impaired dopaminergic reward and cognitive control systems, other brain networks may also be altered in IUD.

One possible candidate is the language system of the brain. Language processing is a unique ability of humans. Evaluating and mapping this function of the brain is critical not only for planning neurosurgery in neurological patients [8] but also for understanding some psychiatric conditions, including autism [9] and schizophrenia [10]. To our knowledge, no one has investigated language networks in addictions with neuroimaging techniques, however, indirect evidences suggest their involvement. Firstly, language performance decline was found in some behavioral and drug addiction forms, including gambling [11], alcoholism [12], tobacco smoking [13], and cannabis use [14]. To our knowledge, only one study investigated verbal skills in generalized IUD. Accordingly, Nie et al. [15] reported significantly poorer language performance in a severe IUD group. They also claimed that it is impossible to decide whether the existing psychological (cognitive) factors were the causes for IUD or if the reverse was true. Secondly, some resting-state fMRI studies revealed functional components that contain brain areas strongly related to language processing in IUD (for a review, see [7]. For example, Liu et al. [16] found regional homogeneity abnormalities in an IUD group in the left superior frontal gyrus (related to language switching [17], right inferior frontal gyrus (involved in verbal discrimination [18]), left superior temporal and middle temporal gyrus (involved in phonological and semantic processing [19]).

Recent studies highlighted that besides the “*core language network*” several additional brain networks are involved in proper language performance and communication. These networks include the *theory of mind system* [20] (temporo-parietal junction, temporal lobe, medial frontal cortex and the precuneus), *visual areas* [21] (several occipital regions, that play crucial role in semantic processing), *motor and sensorimotor areas* [22] (that provides motor control over speech production and includes e.g. the primary motor cortex and supplementary motor area) and *areas related to cognitive control* [23] (prefrontal cortex, anterior and posterior cingulate cortex and angular gyrus) (for review see [24]). The theory of mind abilities [25], visual system [26] and cognitive control functions [27] were all found to be altered in internet-related addictions, therefore we expect significant brain alterations in these areas.

Verbal fluency tasks are the most frequently used tools for assessing language performance including lexical knowledge, lexical retrieval ability, and executive control ability [28]. They are often included in clinical neuropsychological assessments and their reliability confirmed by the Wada test [29]. The two commonly used types of fluency tasks are semantic and phonemic. In phonemic fluency task participants has to say as many words as they can beginning with a specific letter, while in semantic fluency task participants are required to say as many words as possible within a previously defined semantic category (e.g. animals, fruits). However, while previous studies used merely semantic fluency tasks we decided to use the phonemic version in our study to explore functional language networks for the following reasons: i) phonemic fluency was found to be more reflective of executive control abilities [28]–that are also altered in IUD [30]; ii) phonemic fluency showed higher activation than semantic fluency in the frontal lobe [31]. This indicates that psychophysiological interaction analysis (PPI) with Broca’s area seed may reveal more pronounced and reliable functional connections through this task.

In this study, our aim was to investigate IUD-related language network alterations in young adults using fMRI during a phonemic fluency task. To get a clear picture about the phenomenon, blood-oxygen-level-dependent (BOLD) based activation analysis and PPI analysis were performed.

## Materials and methods

### Participants

Sixty healthy right-handed Caucasian university students (30 males) aged between 18 and 30 [mean ± standard deviation (SD): 22.0 ± 2.08 years] were selected from a total of 602 adults, who participated in an online survey. All selected participants underwent a brief interview by a clinical expert (GD) to screen them in relation to a current psychiatric disorder. According to the Edinburg Handedness Inventory [32], all subjects had right-hand dominance and were paid for their participation. According to their oral report after MRI measurements, subjects were naive regarding the purpose of the experiment. The study procedures were carried out in accordance with the Declaration of Helsinki. The board of the Local Ethical Review Committee of the Faculty of Medicine approved the study. All subjects were informed about the study and all provided written informed consent.

### Measures

IUD was assessed using the Problematic Internet Use Questionnaire (PIUQ) [2]. The questionnaire consists of 18 items and three subscales: obsession, neglect, and control disorder. The obsession subscale contains six items concerning obsessive thinking about the Internet and withdrawal symptoms (depression, worry) caused by a lack of Internet use. The six items of the neglect subscale refer to neglect of everyday activities and social life due to Internet use. The control disorder subscale measures the inability to control Internet use with six items. All items were answered on a five-point Likert-type scale (never, rarely, sometimes, often, and always). The three subscales add up to the total score, where a higher score signifies a higher level of addiction. Owing to the lack of well-established diagnostic criteria, we decided to use a multidimensional continuous measure of IUD. The test has good psychometric properties with a Cronbach’s alpha of 0.87. To assess subjective rating of addiction and subjective estimation of overall Internet use, two additional items were inserted into the questionnaire package: Q1. “To what extent are you addicted to the Internet? Indicate on a 0–100 scale”; Q2. “How much time do you spend on the Internet each day? Indicate in hours/day”.

We also assessed individual levels of depression and anxiety. Depression was measured using the Beck Depression Inventory (BDI) [33], while the trait part of the State-Trait Anxiety Inventory (STAI-T) [34] served as an indicator of trait anxiety.

### Functional MRI stimulation paradigm

A block design phonemic verbal fluency task was used to investigate neural correlates of language processing. The paradigm included seven cycles of a 30-second-long rest alternating with a 30-second-long internal word generation task (r-A-r-A-r-A-r-A-r-A-r-A-r-A; where r = rest and A = active phases). During the active conditions, the subjects were asked to silently generate different words in Hungarian starting with a particular letter without any movements (e.g. pronunciation) until the word “end” was announced indicating the rest phase. During the rest periods, they were instructed to stop the active task and relax. The seven different starting letters (S, K, E, T, L, A, N) were presented via MRI-compatible electrostatic headphones specifically designed for fMRI (NordicNeuroLab, Bergen, Norway). Subjects laid in the scanner quietly with their eyes closed during both conditions. The whole fMRI examination was explained in detail before scanning and subjects were questioned to ensure the instructions were fully understood. Owing to the lack of behavioral measures–that would have led to movement artifacts–a visual check of mean activation patterns was needed to ensure that the participants followed our instructions. All participants were involved in the task.

### MRI data acquisition

Subjects were imaged using a 3T Magnetom Tim TRIO, human whole-body MRI scanner with a 12-channel head coil.

Functional images were obtained using a standard 2D echo planar imaging (EPI) sequence with the following parameters: Repetition time (TR)/Echo time (TE) = 2000/36 ms; Flip angle (FA) = 76°; Field of view (FOV) = 230 x 230 mm^2^; 92 x 92 matrix; 23 axial slices with a thickness of 4 mm, 1358 Hz/pixel receiver bandwidth, interleaved slice order.

After the fMRI measurement, field mapping sequence (TR/TE1/TE2 = 400/4.92/7.38 ms; FA = 60°; 36 axial slices; FOV = 228 × 228 mm^2^; 76 × 76 matrix; receiver bandwidth = 259 Hz/pixel) with the same voxel size, orientation, and adjustment parameters as the fMRI scan was used for distortion correction.

Anatomical images were acquired using a T1-weighted axial 3D MPRAGE sequence (TR/TE/Inversion time = 1900/3.41/900 ms; FA = 9°; FOV = 210 x 240 mm^2^; 224 x 256 matrix; slice thickness = 0.94 mm; 160 slices, 180 Hz/pixel receiver bandwidth).

### fMRI data preprocessing

The same preprocessing steps were applied in brain activation analysis and functional connectivity (FC) analysis.

Preprocessing steps, activation analysis, and PPI analysis were performed using FMRIB Software Library (FSL) v6.0, a freely available software tool (www.fmrib.ox.ac.uk/fsl). Preprocessing consisted of removal of nonbrain structures using the Brain Extraction Tool [35], motion correction by MCFLIRT [36], spatial smoothing (Gaussian kernel, 5 mm full width at half maximum), EPI distortion correction with FSL FUGUE [37], and high-pass temporal filtering with a 100 s cutoff.

The functional images were registered into the MNI152 standard space in two steps [38]. Firstly, a functional image of each subject was registered to that subject’s T1 structural scan using boundary based registration (BBR) (6 degrees of freedom). Then, each subject’s anatomical T1 image was registered to the 2 x 2 x 2 mm MNI152 standard space T1 image using a 12 degrees of freedom linear fit followed by nonlinear registration (warp resolution = 10 mm). These two processes were then combined and applied to the first-level statistical maps to take them into standard space.

### Task-related brain activation analysis

Whole brain general linear model time-series statistical analysis of individual data sets were carried out using FILM (FMRIB’s Improved Linear Model) with local autocorrelation correction. The subject level mean activation during word generation was obtained by running FSL first-level analysis. Higher-level mixed-effects analysis were carried out using FLAME 1 (FMRIB’s Local Analysis of Mixed Effects, stage 1) with outlier de-weighting to investigate the following questions: positive and negative correlation analysis between questionnaire data and BOLD signal change during word generation task. In higher-level analysis, BDI and STAI-T scores were used as covariates.

Statistical maps were considered to be significant at Z > 3.1 and a corrected cluster significance threshold of p = 0.05. Since previous findings indicated that brain areas with constantly decreasing activity during demanding tasks are considered part of the default mode network (DMN) [39], deactivated regions indicate DMN-related voxels in the brain.

### Task-related brain functional connectivity analysis

To determine FC, we performed a whole brain PPI analysis in FSL using the seed region in Broca’s area. PPI analysis simply detects task-specific changes in the relationship between the seed region and other voxels within the brain.

Our seed was structurally and functionally constrained:

1. As a first step, cortical reconstruction and segmentation were performed on the T1-weighted anatomical images using Freesurfer 6.0 (https://surfer.nmr.mgh.harvard.edu/fswiki) [40]. Then, visual quality control was performed. When we found errors during the reconstruction, error correction was used according to the recommended workflow

(http://surfer.nmr.mgh.harvard.edu/fswiki/RecommendedReconstruction). The automatic cortical parcellation was based on Freesurfer’s Desiken-Killiany atlas. The anatomical labels of Broca’s area (including the left pars triangularis and left pars opercularis) were extracted and merged for each subject separately.

2. These subject-specific regions of interest (ROIs) on T1-weighed images were then transformed onto the subject’s functional space through BBR linear registration with six degrees of freedom using FSL FLIRT.

3. To ensure that only significantly activated voxels were included in the final ROIs, on an individual level, we excluded voxels that did not show significant activations during the task (Z < 2.3).

4. We estimated and extracted average time courses within our subject-specific Broca’s area ROIs.

The PPI analysis on the first level were conducted according the pipeline suggested by FSL experts (https://fsl.fmrib.ox.ac.uk/fsl/fslwiki/PPIHowToRun) [41]. Second-level mixed-effects analysis was carried out using FLAME 1 (FMRIB’s Local Analysis of Mixed Effects, stage 1) with outlier de-weighting to investigate the following questions: correlations between questionnaire data and PPI. In the higher-level analysis, BDI and STAI-T scores were used as covariates. Similar to the task-based fMRI analysis, statistical maps were considered significant at Z > 2.3 and a corrected cluster significance threshold of p = 0.05.

## Results

### Questionnaire data

The descriptive statistics (median, minimum, and maximum values) are reported in Table 1.

**Table 1:** Descriptive statistics for questionnaire data.

	PIUQ control	PIUQ neglect	PIUQ obsession	PIUQ total	Perceived addiction severity (Q1)	Time spent on the Internet (Q2)	BDI	STAI-T
Median	11	11	10	34	40	120	6.5	39
Min–Max	6–22	6–24	6–20	18–63	0–100	1–857	0–28	22–71

PIUQ = Problematic Internet Use Questionnaire.

Q1 = additional question [“To what extent are you addicted to the Internet?” (The scores are reported on a 0–100 scale)].

Q2 = additional question [“How much time do you spend on the Internet each day?” (The scores are reported in min/day)].

BDI = Beck Depression Inventory.

STAI-T = State-Trait Anxiety Inventory.

### Task-related brain activation analysis

**Fig 1:**
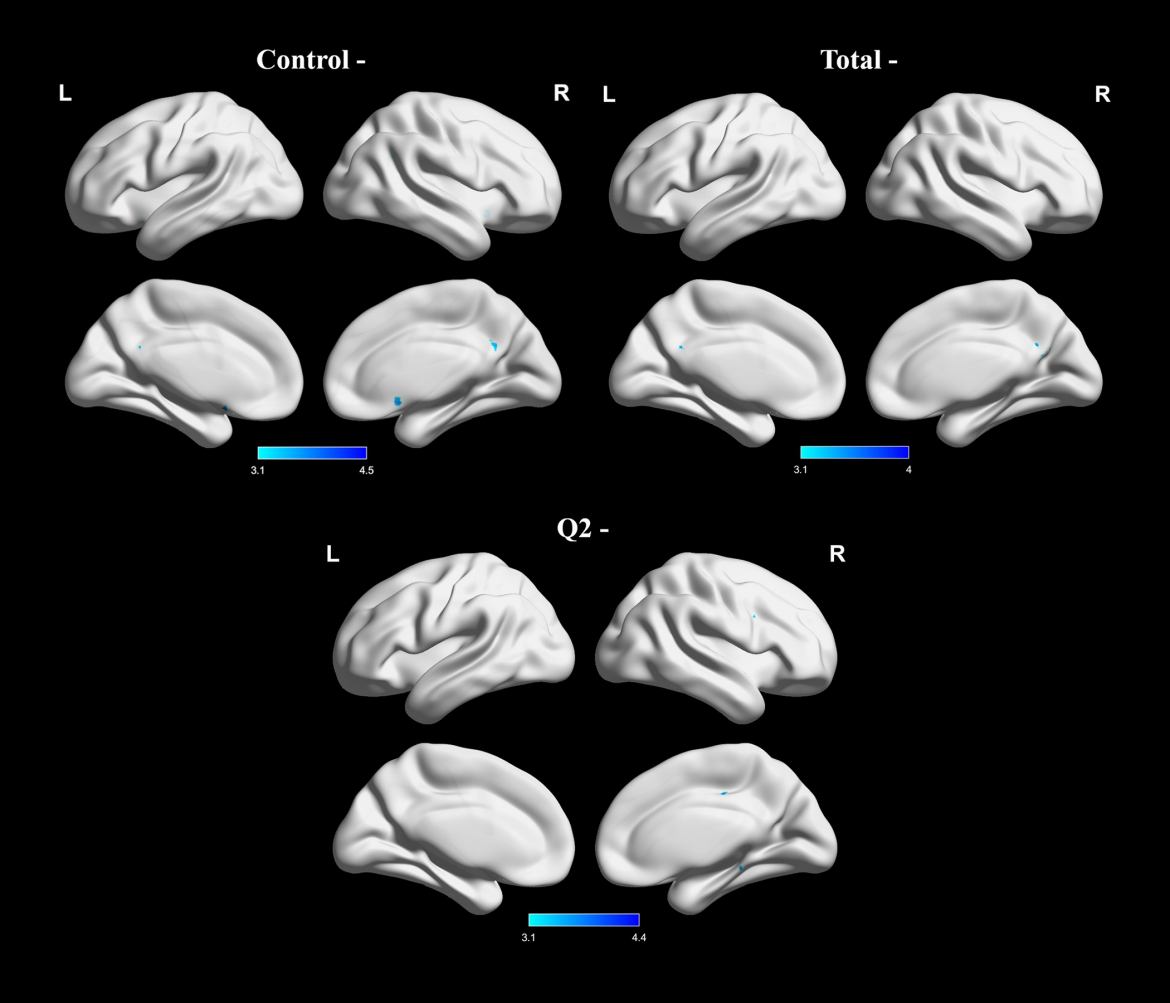
Group level negative associations between BOLD signal changes during verbal fluency task and PIUQ scores (control and total) or Q2 scores (“How much time do you spend on the Internet each day”). Images were thresholded using clusters determined by Z > 3.1 and a corrected cluster significance threshold of p  =  0.05. Light blue–dark blue colors indicate negative correlations.

The correlations between questionnaire scores and BOLD fMRI results during the verbal fluency task are presented in Table 2 (see also Fig 1). The PIUQ control subscale negatively correlated with deactivations in the bilateral precuneus, bilateral posterior cingulate gyrus (PCC), left subcallosal cortex, and left orbitofrontal cortex. The PIUQ total score negatively correlated with bilateral precuneus and bilateral PCC deactivations. The subjective report of Internet usage (Q2) was negatively correlated with deactivations in the right thalamus, right parahippocampal gyrus, and right PCC, while question rates that indicated subjective addiction levels (Q1) did not show significant results. The mean group activation map is available as a (S1 Fig). The results of the voxel-based correlation analyses are also available as a (S1 File).

**Table 2:** Brain areas displaying a correlation between questionnaire scores and BOLD responses during the verbal fluency task.

NC	k	H	Area	Activation/ Deactivation	Max. Z-score	MNI coordinates		
						x	y	z
		PIUQ control subscale						
1	241	bilat	Precuneus ^N^	D*	4.03	0	−62	18
			Posterior cingulate cortex^N^					
2	133	L	Subcallosal cortex^N^	D*	4.48	−12	12	−16
			Orbitofrontal cortex^N^					
		PIUQ neglect subscale						
		NS						
		PIUQ obsession subscale						
		NS						
		PIUQ total						
1	242	bilat	Precuneus^N^	D*	3.97	12	−66	24
			Posterior cingulate cortex^N^					
		Q1 (“To what extent are you addicted to the Internet?”)						
		NS						
		Q2 (“How much time do you spend on the Internet each day?”)^h^						
1	150	R	Thalamus^N^	D*	4.17	10	−34	6
		R	Parahippocampal gyrus^N^	D	3.86	18	−34	−8
2	108	R	Posterior cingulate cortex^N^	D*	3.87	8	−22	42

NC = number of clusters in a given contrast.

k = number of voxels in each cluster.

H = hemisphere.

R = right hemisphere.

L = left hemisphere.

^N^ negative correlation between BOLD responses and questionnaire scores.

A = activation.

D = deactivation.

NS = no significant results.

* significant group mean activation or deactivation (Z > 2.3).

MNI coordinates in mm are listed for the local maxima; brain areas were identified according to the Harvard–Oxford cortical and subcortical atlases.

### Task-related brain FC analysis

**Fig 2:**
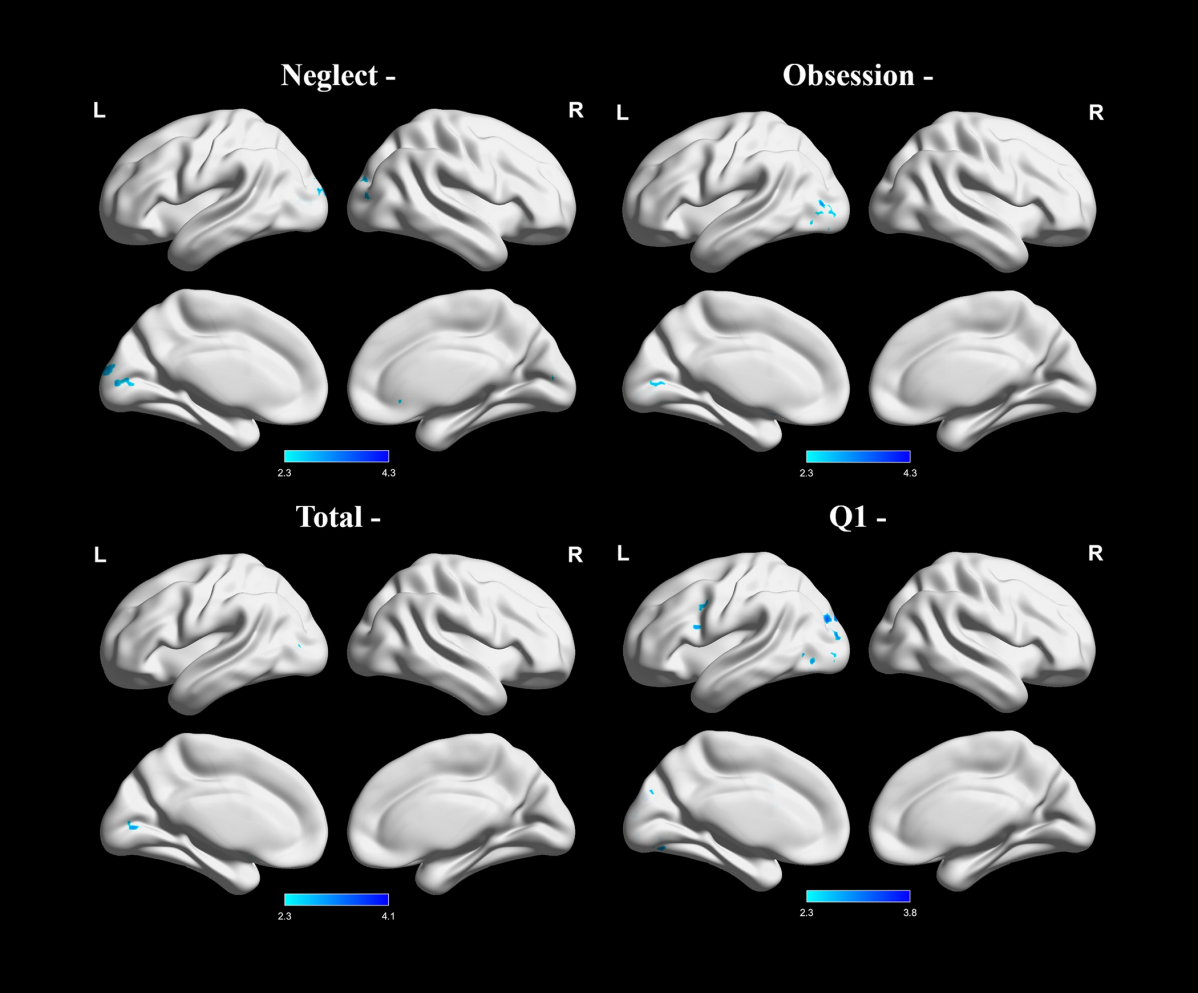
Group level negative associations between PIUQ neglect, obsession, total scores, and Q1 (“To what extent are you addicted to the Internet?”) score and the results of psychophysiological interaction analysis. Images were thresholded using clusters determined by Z > 2.3 and a corrected cluster significance threshold of p = 0.05. Light blue–dark blue colors indicate negative correlations.

As described earlier, Broca’s area was used as the seed region in PPI analysis. Negative correlations were found between the PIUQ neglect subscale and PPI in the left occipital pole, left lateral occipital cortex (LOC), and left intracalcarine cortex. The PIUQ obsession was negatively associated with PPI in the left LOC, intracalcarine cortex accumbens and caudate nucleus, while PIUQ total was also negatively correlated with the left caudate nucleus, LOC and intracalcarine cortex. Question rates that indicated subjective addiction levels (Q1) showed negative association with the PPI in the left occipital pole, LOC, occipital fusiform gyrus, middle frontal gyrus and precentral gyrus (Table 3, Fig 2). The results of the PPI analyses (in NIFTI format) are available as a (S1 File).

**Table 3:** Association between questionnaire scores and functional connectivity with Broca’s area for the verbal fluency task.

NC	k	H	Area	Max. Z-score	MNI coordinates		
					x	y	z
		PIUQ control subscale					
		NS					
		PIUQ neglect subscale					
1	1720	L	Occipital pole^N^	4.19	-12	-94	16
			Lateral occipital cortex^N^ Intracalcarine cortex^N^				
		PIUQ obsession subscale					
1	719	L	Lateral occipital cortex^N^ Intracalcarine cortex^N^	4.29	-36	-78	6
2	350	L	Accumbens^N^	3.1	-8	18	-2
			Caudate nucleus^N^				
		PIUQ total					
1	349	L	Caudatue nucleus^N^	2.66	-16	24	-2
2	303	L	Lateral occipital cortex^N^	3.56	-36	-78	6
		L	Intracalcarine cortex^N^	2.99	-10	-76	4
		Q1 (“To what extent are you addicted to the Internet?”)					
1	748	L	Occipital pole^N^	3.71	-14	-94	32
			Lateral occipital cortex^N^				
2	544	L	Lateral occipital cortex^N^	3.66	-32	-84	-8
			Occipital Fusiform Gyrus^N^				
3	302	L	Middle Frontal Gyrus^N^	3.82	-48	10	40
			Precentral Gyrus^N^				
		Q2 (“How much time do you spend on the Internet each day?”)					
		NS					

NC = number of clusters in a given contrast.

k = number of voxels in each cluster.

H = hemisphere.

L = left hemisphere

^N^ negative correlation between BOLD responses and questionnaire scores.

NS = no significant results.

MNI coordinates in mm are listed for the local maxima; brain areas were identified according to the Harvard–Oxford cortical and subcortical atlases.

## Discussion

In the current study, we investigated brain activations and functional brain connections of Broca’s area during a verbal fluency task. We used conventional BOLD activation and PPI analysis and demonstrated altered language processing in IUD. According to our results, alterations in the language system manifested in two different relations: altered DMN functions during a verbal fluency task and altered functional connections between Broca’s and visual brain areas.

In this study, we found a negative correlation between deactivations of brain areas that are considered key components in the DMN (precuneus and PCC) and control plus total score of PIUQ. The involvement of the DMN-related areas in IUD was not unexpected. In a previous study, BOLD signal changes were found to be negatively correlated with PIUQ in precuneus and PCC during the Stroop task [42]. There, we claimed that these findings may indicate a long-term negative effect of IUD on cognition–similar to that presented in other addiction forms. We also suggested that DMN alterations serve as the common neural background behind cognitive deficits in IUD. Our current findings support this theory: beside a demanding attention task that requires cognitive control (Stroop task), negative associations between IUD and activations in precuneus and PCC are also present during the language task. However, as it will be elucidated later, it is not decided yet whether these alterations are specific for language processing and cognitive control processes or are part of a general cognitive decline.

Important findings were also related to the occipital areas of the brain. The PIUQ neglect subscale showed a negative correlation with FC between the Broca’s region and left lateral occipital cortex (LOC), occipital pole and intracalcarine cortex, the PIUQ obsession and total scores were inversely related to FC of Broca’s region and right LOC and intracalcarine cortex, while Q1 (the extent of internet addiction) was again inversely correlated with FC in the occipital pole, LOC and occipital fusiform gyrus. The involvement of the occipital cortex in word production is well understood since it is often implicated in neuroimaging studies of naming and object recognition [43, 44]. Additionally, Broca’s connectivity with occipital areas highlights that regions involved in visual processing seem to be crucial for verbal fluency [45]. Furthermore, FC most probably arises from structural connectivity of the left inferior frontal areas, particularly the inferior fronto-occipital fasciculus (IFOF). This tract runs ventrally between the frontal and the temporo-occipital regions and has been implicated in, among others, the semantic processing of language [46]. Possibly, the most convincing evidence for the involvement of IFOF in semantic processing comes from brain stimulation studies, e.g. Martino and colleagues found that intraoperative electrical stimulation of the IFOF induces semantic paraphasias (errors with regard to the meaning of the target word) [47]. Therefore, our results suggest that the semantic system is also impaired in IUD, but without reliable behavioral evidence, this explanation seems less justified.

Our results indicate that two different effects are present. Firstly, we can assume that there is a direct association between problematic Internet use and language function independent of other cognitive domains. Alterations in the FC including language specific regions such as the Broca’s area are unlikely to be the outcome of deficits in other cognitive domains (e.g. attention) or just a secondary outcome of psychological symptoms that have been reported in IUD (e.g. depression and anxiety). We assume that less personal social interactions, new communication forms, or the breaking through of the audio-visual technology may be the main reasons but given the lack of psychological research on the topic, these assumptions must be treated as speculations. The second effect is the general cognitive decline induced by constant regulation of craving. In our case, craving can be defined as a constant intense urge to use the Internet for nonspecific purposes that make users feel pleasure [48]. According to the cognitive processing model, nonautomatic processes are activated to prevent the execution of automatized behaviors that are constantly present in addictions and are initiated by specific cues. When a person wants to prevent or stop executing these problematic behavioral acts, cognitive effort is required. This effort can exhaust limited cognitive resources, that may result in a decrease of cognitive performance in everyday life [49]. If altered language processing is part of a general cognitive decline, that is related to constant regulation of craving, two assumptions need to be satisfied: firstly, other cognitive domains should also be altered in IUD. Previous studies showed that generalized or specific forms of IUD are related to reduced inhibition [50], attention deficit and impulsivity [51], working memory and executive functions [52]. The second assumption is that if craving symptoms decrease (e.g. after successful treatment), cognitive symptoms should also diminish. Unfortunately, treatment-induced cognitive outcomes in IUD have not been investigated yet, although positive effects were described in substance addictions: after a short abstinence interval (three months), chronic methamphetamine users showed improved performance in motor and verbal memory tests [53].

Some limitations must be considered. The most significant is the lack of behavioral data regarding performance in the verbal fluency task. Although one previous study revealed impaired verbal fluency in IUD, without behavioral data, uncertainty surrounds the interpretation of the fMRI results. Secondly, a semantic verbal fluency and comprehension task should also be used in the future. They would make our results more comparable with previous behavioral and neuroscientific findings and we would get much clearer picture about the IUD-related language impairments. The cross-sectional nature of the study limits our ability to discriminate between cause and effect. It is impossible to decide if alterations in the brain’s language system lead to IUD or vice versa. To get a clearer picture concerning the development of language problems, longitudinal studies–merely on an adolescent sample–are highly needed. It is also under debate, which exact online behaviors lead to language system alterations. Therefore, in the future, a second study with an extended questionnaire battery and resting-state fMRI measurement is needed to answer this question.

## Conclusions

Taken together our findings suggest that IUD in young adults is related to functional brain changes in language areas. These finding may address the neural background of decreased verbal fluency performance reported previously in the literature. According to our results, two main neural systems may be altered: the DMN and the FC between Broca’s area and the occipital lobe areas. However, it is important to note that without behavioral data and well-established longitudinal studies, these results should be interpreted carefully. Nevertheless, as we know, this is the very first study to investigate IUD-related alterations in the language system, and our results emphasize that beside the brain’s reward and inhibitory control systems, the language system is the next candidate to be involved in the pathogenesis of IUD.

## Supporting information

S1 Fig Mean group activation maps during verbal fluency task. Color-coding indicates the Z-value.
(TIF)

S1 File The results of the voxel-based correlation analyses in NIFTI. ACT–activation analysis, PPI—psychophysiological interaction analysis.
(7Z) Click here for additional data file.

## References

[1] Young KS. Internet Addiction: The Emergence of a New Clinical Disorder. CyberPsychology Behav. 1998;1: 237–244. doi: 10.1089/cpb.1998.1.237

[2] Demetrovics Z, Szeredi B, Rózsa S. The three-factor model of Internet addiction: The development of the Problematic Internet Use Questionnaire. Behav Res Methods. 2008;40: 563–574. doi: 10.3758/brm.40.2.563 18522068

[3] Kuss DJ, Lopez-Fernandez O. Internet addiction and problematic Internet use: A systematic review of clinical research. World J Psychiatry. 2016;6: 143. doi: 10.5498/wjp.v6.i1.143 27014605 PMC4804263

[4] Montag C, Bey K, Sha P, Li M, Chen Y-FF, Liu W-YY, et al. Is it meaningful to distinguish between generalized and specific Internet addiction? Evidence from a cross-cultural study from Germany, Sweden, Taiwan and China. Asia-Pacific Psychiatry. 2015;7: 20–26. doi: 10.1111/appy.12122 24616402

[5] Dong G, DeVito EE, Du X, Cui Z. Impaired inhibitory control in ‘internet addiction disorder’: A functional magnetic resonance imaging study. Psychiatry Res Neuroimaging. 2012;203: 153–158. doi: 10.1016/j.pscychresns.2012.02.001 22892351 PMC3650485

[6] Dong G, Huang J, Du X. Enhanced reward sensitivity and decreased loss sensitivity in Internet addicts: An fMRI study during a guessing task. J Psychiatr Res. 2011;45: 1525–1529. doi: 10.1016/j.jpsychires.2011.06.017 21764067

[7] Sepede G, Tavino M, Santacroce R, Fiori F, Salerno RM, Di Giannantonio M. Functional magnetic resonance imaging of internet addiction in young adults. World J Radiol. 2016;8: 210. doi: 10.4329/wjr.v8.i2.210 26981230 PMC4770183

[8] Tanaka N, Stufflebeam SM. Presurgical mapping of the language network using resting-state functional connectivity. Top Magn Reson Imaging. 2016;25: 19–23. doi: 10.1097/RMR.0000000000000073 26848557 PMC4833007

[9] Liu J, Tsang T, Jackson L, Ponting C, Jeste SS, Bookheimer SY, et al. Altered Lateralization of Dorsal Language Tracts in 6-Week-Old Infants at Risk for Autism. Dev Sci. 2018; e12768. doi: 10.1111/desc.12768 30372577 PMC6470045

[10] Cavelti M, Kircher T, Nagels A, Strik W, Homan P. Is formal thought disorder in schizophrenia related to structural and functional aberrations in the language network? A systematic review of neuroimaging findings. Schizophr Res. 2018;199: 2–16. doi: 10.1016/j.schres.2018.02.051 29510928

[11] Conversano C, Marazziti D, Carmassi C, Baldini S, Barnabei G, Dell’Osso L. Pathological gambling: A systematic review of biochemical, neuroimaging, and neuropsychological findings. Harv Rev Psychiatry. 2012;20: 130–148. doi: 10.3109/10673229.2012.694318 22716504

[12] Réka M, Oguz K, Tamás S, Dezsõ N. Cognitive impairment in patients with alcoholism after long-term abstinence. Neuropsychopharmacol Hungarica. 2009;11: 135–139. Available: https://europepmc.org/abstract/med/20128392 20128392

[13] Núñez C, Stephan-Otto C, Cuevas-Esteban J, Maria Haro J, Huerta-Ramos E, Ochoa S, et al. Effects of caffeine intake and smoking on neurocognition in schizophrenia. Psychiatry Res. 2015;230: 924–931. doi: 10.1016/j.psychres.2015.11.022 26614014

[14] Blanco-Hinojo L, Pujol J, Harrison BJ, Macià D, Batalla A, Nogué S, et al. Attenuated frontal and sensory inputs to the basal ganglia in cannabis users. Addict Biol. 2017;22: 1036–1047. doi: 10.1111/adb.12370 26934839

[15] Nie J, Zhang W, Liu Y. Exploring depression, self-esteem and verbal fluency with different degrees of internet addiction among Chinese college students. Compr Psychiatry. 2017;72: 114–120. doi: 10.1016/j.comppsych.2016.10.006 27810547

[16] Liu J, Gao XP, Osunde I, Li X, Zhou SK, Zheng H rong, et al. Increased regional homogeneity in internet addiction disorder: A resting state functional magnetic resonance imaging study. Chin Med J (Engl). 2010;123: 1904–1908. doi: 10.3760/cma.j.issn.0366-6999.2010.14.014 20819576

[17] Wang H, Chen H, Liu W, Li J, Hou Y. Neural bases of asymmetric language switch in second-language learners: An ERP study. 2009 WRI World Congr Comput Sci Inf Eng CSIE 2009. 2009;7: 239–242. doi: 10.1109/CSIE.2009.733

[18] Hsieh L, Gandour J, Wong D, Hutchins GD. Functional heterogeneity of inferior frontal gyrus is shaped by linguistic experience. Brain Lang. 2001;76: 227–252. doi: 10.1006/brln.2000.2382 11247643

[19] Démonet JF, Chollet F, Ramsay S, Cardebat D, Nespoulous JL, Wise R, et al. The anatomy of phonological and semantic processing in normal subjects. Brain. 1992;115: 1753–1768. doi: 10.1093/brain/115.6.1753 1486459

[20] van Ackeren MJ, Casasanto D, Bekkering H, Hagoort P, Rueschemeyer SA. Pragmatics in action: indirect requests engage theory of mind areas and the cortical motor network. J Cogn Neurosci. 2012;24: 2237–2247. doi: 10.1162/jocn_a_00274 22849399

[21] Fang Y, Han Z, Zhong S, Gong G, Song L, Liu F, et al. The semantic anatomical network: Evidence from healthy and brain-damaged patient populations. Hum Brain Mapp. 2015;36: 3499–3515. doi: 10.1002/hbm.22858 26059098 PMC6869673

[22] Zhang Y, Wang K, Yue C, Mo N, Wu D, Wen X, et al. The motor features of action verbs: fMRI evidence using picture naming. Brain Lang. 2018;179: 22–32. doi: 10.1016/j.bandl.2018.02.002 29501856

[23] Wu J, Yang J, Chen M, Li S, Zhang Z, Kang C, et al. Brain network reconfiguration for language and domain-general cognitive control in bilinguals. Neuroimage. 2019;199: 454–465. doi: 10.1016/j.neuroimage.2019.06.022 31200066

[24] Hertrich I, Dietrich S, Ackermann H. The Margins of the Language Network in the Brain. Front Commun. 2020;5: 93. doi: 10.3389/fcomm.2020.519955

[25] Akdeniz B, Gunduz M, Calli S, Demirdogen E, Yavuz M. Parental attachment and the theory of mind abilities as predictors of internet addiction in Turkish adolescents. Psychiatry Clin Psychopharmacol. 2020;30: 1. doi: 10.5455/pcp.20200427054916

[26] Horvath J, Mundinger C, Schmitgen MM, Wolf ND, Sambataro F, Hirjak D, et al. Structural and functional correlates of smartphone addiction. Addict Behav. 2020;105: 106334. doi: 10.1016/j.addbeh.2020.106334 32062336

[27] Wang Y, Qin Y, Li H, Yao D, Sun B, Li Z, et al. Abnormal Functional Connectivity in Cognitive Control Network, Default Mode Network, and Visual Attention Network in Internet Addiction: A Resting-State fMRI Study. Front Neurol. 2019;10: 1006. doi: 10.3389/fneur.2019.01006 31620077 PMC6759465

[28] Shao Z, Janse E, Visser K, Meyer AS. What do verbal fluency tasks measure? Predictors of verbal fluency performance in older adults. Front Psychol. 2014;5: 772. doi: 10.3389/fpsyg.2014.00772 25101034 PMC4106453

[29] Woermann FG, Jokeit H, Luerding R, Freitag H, Schulz R, Guertler S, et al. Language lateralization by Wada test and fMRI in 100 patients with epilepsy. Neurology. 2003;61: 699–701. doi: 10.1212/01.wnl.0000078815.03224.57 12963768

[30] Dong G, Zhou H, Zhao X. Male Internet addicts show impaired executive control ability: Evidence from a color-word Stroop task. Neurosci Lett. 2011;499: 114–118. doi: 10.1016/j.neulet.2011.05.047 21645588

[31] Heim S, Eickhoff SB, Amunts K. Specialisation in Broca’s region for semantic, phonological, and syntactic fluency? Neuroimage. 2008;40: 1362–1368. doi: 10.1016/j.neuroimage.2008.01.009 18296070

[32] Oldfield RC. The assessment and analysis of handedness: The Edinburgh inventory. Neuropsychologia. 1971;9: 97–113. doi: 10.1016/0028-3932(71)90067-4 5146491

[33] Beck AT, Steer RA, Carbin MG. Psychometric properties of the Beck Depression Inventory: Twenty-five years of evaluation. Clin Psychol Rev. 1988;8: 77–100. doi: 10.1016/0272-7358(88)90050-5

[34] Spielberger CD, Gonzalez-Reigosa F, Martinez-Urrutia A, Natalicio LFS, Natalicio DS. The State-Trait Anxiety Inventory. Rev Interam Psicol J Psychol. 2017;5. doi: 10.30849/RIP/IJP.V5I3&amp;4.620

[35] Jenkinson M, Pechaud M, Smith S. BET2-MR-Based Estimation of Brain, Skull and Scalp Surfaces. Human Brain Mapping. 2002. pp. 143–155. Available: www.fmrib.ox.ac.uk/analysis/research/bet12391568

[36] Jenkinson M, Bannister P, Brady M, Smith S. Improved optimization for the robust and accurate linear registration and motion correction of brain images. Neuroimage. 2002;17: 825–841. doi: 10.1016/s1053-8119(02)91132-8 12377157

[37] Jenkinson M. Improving the registration of B0-disorted EPI images using calculated cost function weights. Tenth Int Conf on Functional Mapping of the Human Brain 2004. 2004. pp. 143–155.

[38] Jenkinson M, Smith S. A global optimisation method for robust affine registration of brain images. Med Image Anal. 2001;5: 143–156. doi: 10.1016/s1361-8415(01)00036-6 11516708

[39] Shulman GL, Fiez JA, Corbetta M, Buckner RL, Miezin FM, Raichle ME, et al. Common Blood Flow Changes across Visual Tasks: II. Decreases in Cerebral Cortex. J Cogn Neurosci. 1997;9: 648–663. doi: 10.1162/jocn.1997.9.5.648 23965122

[40] Dale AM, Fischl B, Sereno MI. Cortical surface-based analysis: I. Segmentation and surface reconstruction. Neuroimage. 1999;9: 179–194. doi: 10.1006/nimg.1998.0395 9931268

[41] Friston KJ, Buechel C, Fink GR, Morris J, Rolls E, Dolan RJ. Psychophysiological and modulatory interactions in neuroimaging. Neuroimage. 1997;6: 218–229. doi: 10.1006/nimg.1997.0291 9344826

[42] Darnai G, Perlaki G, Zsidó AN, Inhóf O, Orsi G, Horváth R, et al. Internet addiction and functional brain networks: task-related fMRI study. Sci Rep. 2019;9: 1–10. doi: 10.1038/s41598-019-52296-1 31673061 PMC6823489

[43] DeLeon J, Gottesman RF, Kleinman JT, Newhart M, Davis C, Heidler-Gary J, et al. Neural regions essential for distinct cognitive processes underlying picture naming. Brain. 2007;130: 1408–1422. doi: 10.1093/brain/awm011 17337482

[44] Palejwala AH, O’Connor KP, Pelargos P, Briggs RG, Milton CK, Conner AK, et al. Anatomy and white matter connections of the lateral occipital cortex. Surg Radiol Anat. 2020;42: 315–328. doi: 10.1007/s00276-019-02371-z 31734739

[45] Kepinska O, de Rover M, Caspers J, Schiller NO. Connectivity of the hippocampus and Broca’s area during acquisition of a novel grammar. Neuroimage. 2018;165: 1–10. doi: 10.1016/j.neuroimage.2017.09.058 28970145

[46] Duffau H. The anatomo-functional connectivity of language revisited. New insights provided by electrostimulation and tractography. Neuropsychologia. 2008;46: 927–934. doi: 10.1016/j.neuropsychologia.2007.10.025 18093622

[47] Martino J, Brogna C, Robles SG, Vergani F, Duffau H. Anatomic dissection of the inferior fronto-occipital fasciculus revisited in the lights of brain stimulation data. Cortex. 2010;46: 691–699. doi: 10.1016/j.cortex.2009.07.015 19775684

[48] Andrade J, May J, Kavanagh D. Sensory Imagery in Craving: From Cognitive Psychology to New Treatments for Addiction. J Exp Psychopathol. 2012;3: 127–145. doi: 10.5127/jep.024611

[49] Tiffany ST. Cognitive concepts of craving. Alcohol Res Heal. 1999;23: 215–224. Available: http://www.ncbi.nlm.nih.gov/pubmed/10890817 10890817 PMC6760370

[50] Nie J, Zhang W, Chen J, Li W. Impaired inhibition and working memory in response to internet-related words among adolescents with internet addiction: A comparison with attention-deficit/hyperactivity disorder. Psychiatry Res. 2016;236: 28–34. doi: 10.1016/j.psychres.2016.01.004 26778632

[51] Yen J-Y, Yen C-F, Chen C-S, Tang T-C, Ko C-H. The Association between Adult ADHD Symptoms and Internet Addiction among College Students: The Gender Difference. CyberPsychology Behav. 2008;12: 187–191. doi: 10.1089/cpb.2008.0113 19072077

[52] Zhou Z, Zhou H, Zhu H. Working memory, executive function and impulsivity in Internet-addictive disorders: A comparison with pathological gambling. Acta Neuropsychiatr. 2016;28: 92–100. doi: 10.1017/neu.2015.54 26400106

[53] Wang GJ, Volkow ND, Chang L, Miller E, Sedler M, Hitzemann R, et al. Partial Recovery of Brain Metabolism in Methamphetamine Abusers after Protracted Abstinence. Am J Psychiatry. 2004;161: 242–248. doi: 10.1176/appi.ajp.161.2.242 14754772

