# Supplementary figures and images for: Language processing in Internet use disorder: Task-based fMRI study
Source: PLoS One. 2022 Jun 24;17(6):e0269979. doi: 10.1371/journal.pone.0269979 (PMC9231743; doi:10.1371/journal.pone.0269979)

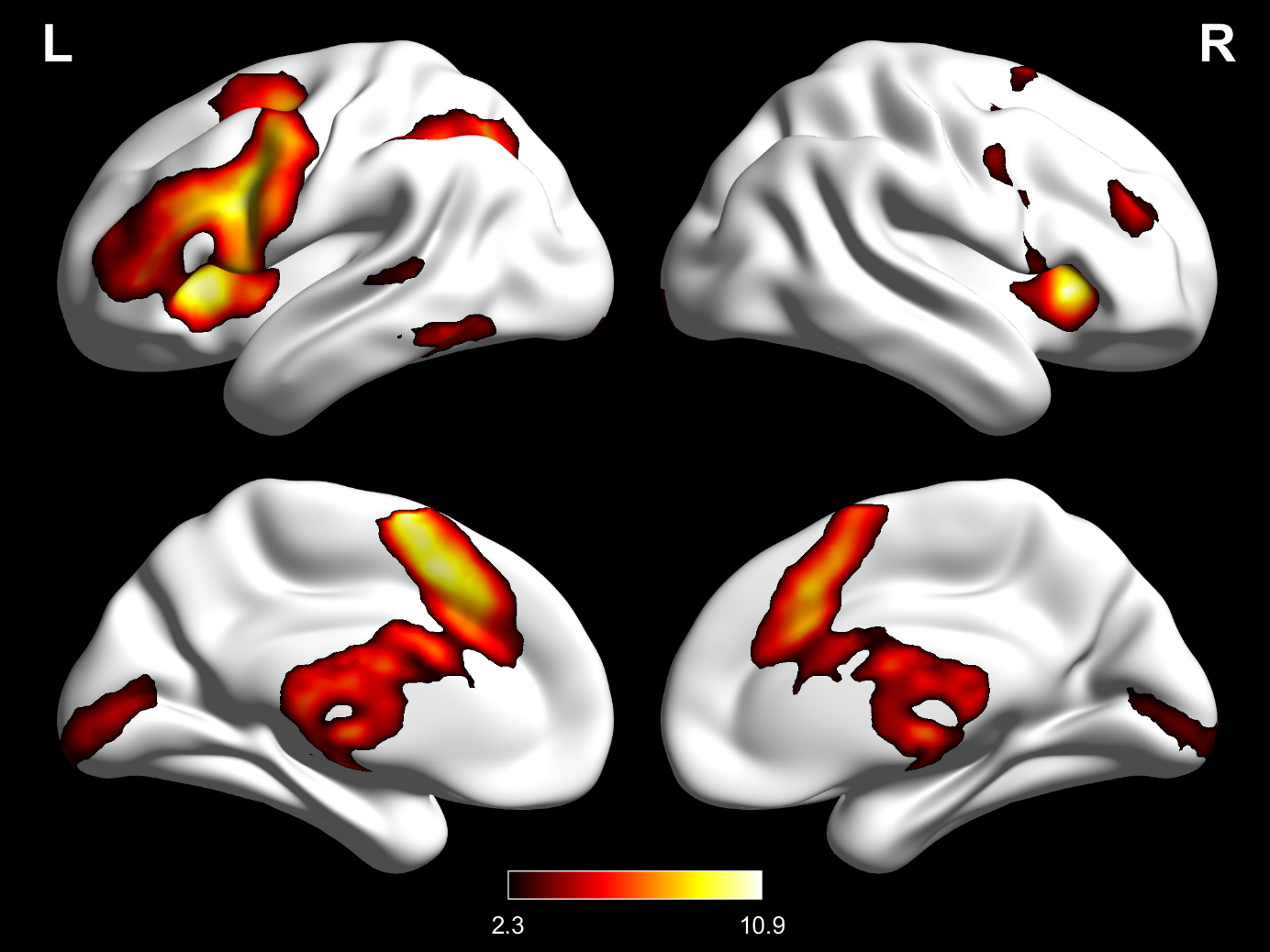

Supplement: S1 Fig — Color-coding indicates the Z-value. (TIF) [file pone.0269979.s001.tif]
